# Supplementary material for: Association between oral microbiome and five types of respiratory infections: a two-sample Mendelian randomization study in east Asian population
Source: Front Microbiol. 2024 Apr 10;15:1392473. doi: 10.3389/fmicb.2024.1392473 (PMC11039966; doi:10.3389/fmicb.2024.1392473)
Supplement: Supplementary file 1 [file Data_Sheet_1.zip › Supplementary Materials/MR plots of saliva/Tonsillitis/s__Prevotella_nanceiensis_mgs_3029/forest.pdf]

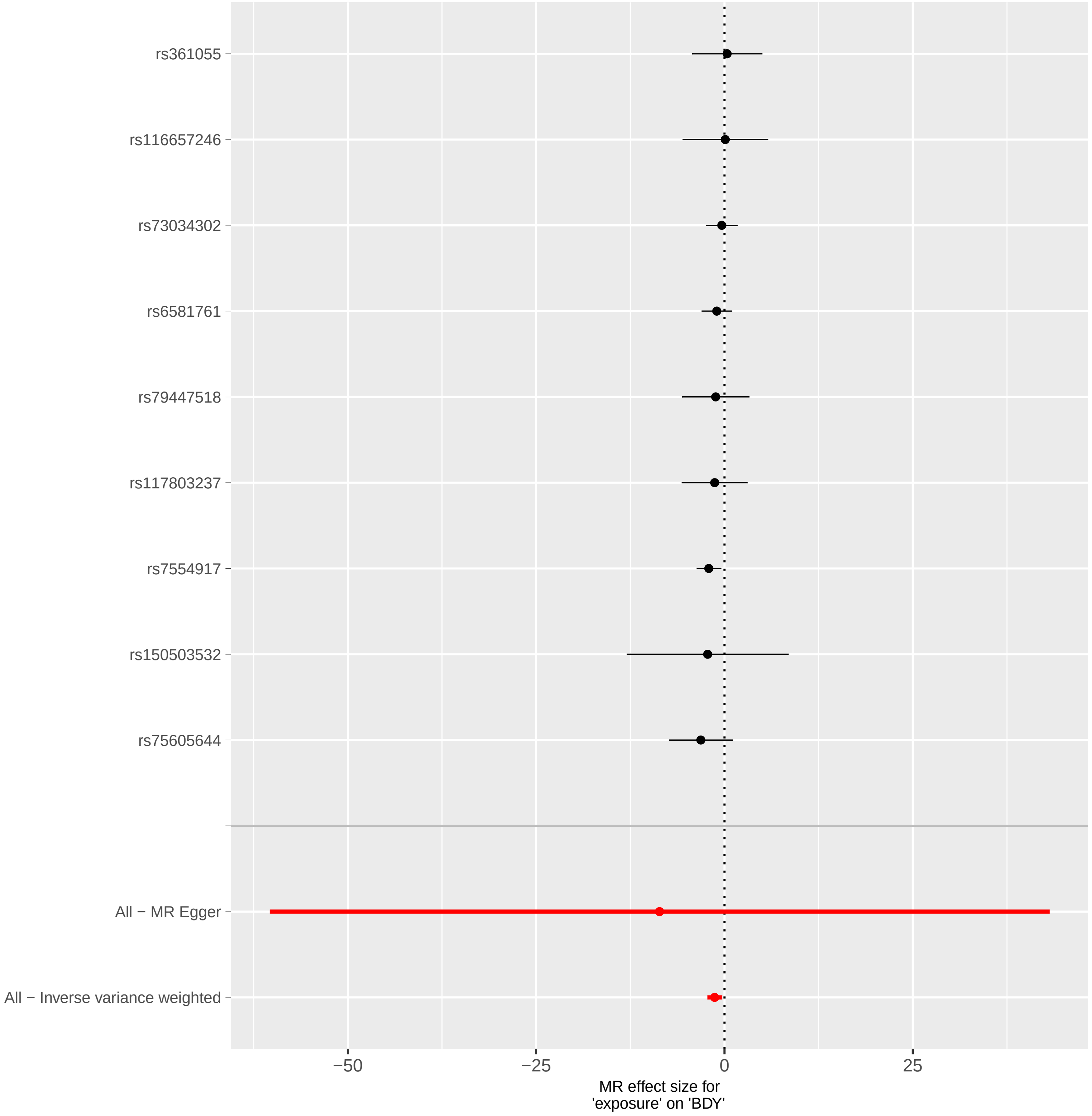
rs361055
rs116657246
rs73034302
rs6581761
rs79447518
rs117803237
rs7554917
rs150503532
rs75605644
All − MR Egger
All − Inverse variance weighted
−50
−25
0
25
MR effect size for
'exposure' on 'BDY'
